# Hemoglobin, Ferritin, and Lactate Dehydrogenase as Predictive Markers for Neonatal Sepsis

**DOI:** 10.3390/jpm14050476

**Published:** 2024-04-29

**Authors:** Nicoleta Lungu, Daniela-Eugenia Popescu, Aniko Maria Manea, Ana Maria Cristina Jura, Florina Marinela Doandes, Zoran Laurentiu Popa, Florin Gorun, Cosmin Citu, Denis Gruber, Sebastian Ciurescu, Marioara Boia

**Affiliations:** 1 Department of Obstetrics-Gynecology and Neonatology, “Victor Babes” University of Medicine and Pharmacy, 300041 Timisoara, Romania; lungu.nicoleta@umft.ro (N.L.); manea.aniko@umft.ro (A.M.M.); doandes.florina@umft.ro (F.M.D.); popa.zoran@umft.ro (Z.L.P.); citu.ioan@umft.ro (C.C.); boia.marioara@umft.ro (M.B.); 2 Doctoral School, ‘Victor Babes’ University of Medicine and Pharmacy, 300041 Timisoara, Romaniadenis.gruber@umft.ro (D.G.); sebastian.ciurescu@umft.ro (S.C.); 3 Department of Obstetrics and Gynecology, Municipal Emergency Clinical Hospital Timisoara, 300172 Timisoara, Romania; gorun.florin@umft.ro

**Keywords:** neonatal sepsis, biomarkers, ferritin, lactate dehydrogenase

## Abstract

(1) Background: This study evaluates the predictive effectiveness of biomarkers in diagnosing newborn sepsis. (2) Methods: This was a case–control study conducted on neonates hospitalized at the Clinical Hospital “Louis Turcanu”, Timisoara, Romania, from October 2018 to July 2023. Using a vacutainer collection device, venous blood was collected at admission for complete blood tests, including ferritin, hemoglobin, LDH, and blood culture analysis. Neonates were divided into two groups: sepsis-positive and sepsis-negative. The outcome of interest was a diagnosis of sepsis. (3) Results: Data from 86 neonates, 51 of whom had been confirmed to have sepsis, were analyzed. This study found no significant difference in gestational age, infant weight, fetal growth restriction, or APGAR score between neonates with and without sepsis. However, there was a higher incidence of sepsis among neonates delivered via cesarean section. Neonatal patients with sepsis showed significantly higher levels of neonatal serum ferritin and LDH compared to those without sepsis. Ferritin and LDH biomarkers demonstrated excellent discriminatory capabilities in diagnosing neonatal sepsis. Logistic regression analysis revealed a significant association between elevated ferritin and LDH levels and the likelihood of neonatal sepsis, while anemia did not show a significant association. (4) Conclusions: LDH and ferritin concentrations are found to be predictive biomarkers for neonatal sepsis, indicating a potential role in detecting susceptible neonates and implementing prompt interventions to improve patient outcomes.

## 1. Introduction

Neonatal sepsis continues to pose a considerable challenge, particularly in middle- and low-income countries, and is an important contributor to morbidity and death rates among newborns [1].

Neonatal sepsis denotes a systemic infection that affects neonates in their first 28 days of life. Neonatal sepsis is classified into two distinct categories, early-onset sepsis (EOS) and late-onset sepsis (LOS), according to the time of presentation following birth. LOS is defined as sepsis that occurs at or after 72 h of life, whereas EOS refers to sepsis in neonates that occurs at or before 72 h of life (although some experts use seven days) [1,2].

Early-onset sepsis (EOS) often occurs when infections are transmitted from the female genitourinary system to the baby or fetus. These bacteria have the ability to go upwards via the vagina, cervix, and uterus, and can even cause infection in the amniotic fluid. Neonates can also acquire infections either in their gestation period or during the birthing process when they traverse the vaginal canal. Maternal conditions that heighten the likelihood of newborn sepsis are chorioamnionitis, GBS infection, preterm birth (before 37 weeks), and protracted rupture of membranes exceeding 18 h [1,3]. Common bacterial infections associated with early-onset sepsis (EOS) include Group B streptococcus (GBS), *Escherichia coli*, coagulase-negative *Staphylococcus*, *Haemophilus influenza*, and *Listeria monocytogenes* [1].

Late-onset sepsis (LOS) typically arises from the transmission of pathogens from the surroundings subsequent to childbirth, including through contact with healthcare personnel or caregivers. The risk of developing LOS is elevated in infants who are required to undergo invasive procedures that disrupt the mucosa, such as intravascular catheter insertion [1]. Coagulase-negative staphylococcal species, particularly *Staphylococcus* epidermis, are the primary cause, accounting for over 50% of cases of late-onset sepsis in developed nations [1].

Neonatal sepsis, particularly in susceptible groups such as those with extremely low birth weight, is highly correlated with higher incidence of preterm complications and increasing neurodevelopmental outcomes [4,5,6,7,8]. Furthermore, the presence of sepsis in conjunction with necrotizing enterocolitis (NEC) was correlated with elevated levels of hemodynamic support, acute kidney damage, and an extended period of postoperative ileus [9]. Moreover, the prevalence of sepsis is notably elevated in preterm newborns, particularly those with a birth weight below 1000 g [1].

A positive blood culture with an identifiable pathogen is considered the definitive method for diagnosing sepsis [8,10].

Sepsis is a multifaceted condition distinguished by the presence of systemic inflammatory response syndrome (SIRS) resulting from either suspected or confirmed infection [11]. Neonatal sepsis represents a significant peril, impacting infants within the neonatal period (less than 28 days) [11]. Although there have been improvements in medical care, newborn sepsis still presents challenges in terms of diagnosis and treatment [2]. It is essential to identify and intervene early on in order to enhance outcomes [2]. However, the diagnostic accuracy of common laboratory measures (such as acute-phase reactants, white blood cell indices, and heart rate characteristics) is constrained due to their low positive predictive value (PPV) in regard to neonatal sepsis [2,12,13,14].

Biomarkers such as ferritin, hemoglobin, and lactate dehydrogenase (LDH) have been investigated in numerous clinical settings, including sepsis in children and adults [15,16,17,18,19,20,21]. Moreover, leukocytes, lymphocytes, and the biomarkers CK, CRP, LDH, AST, ferritin, and IL-6 accurately determine the inflammatory condition of newborns in some infections [22].

Lactate dehydrogenase (LDH) is an enzyme that plays a crucial role in cellular metabolism. Elevated levels of LDH in the bloodstream during sepsis can be attributed to many processes, such as cellular damage caused by bacterial toxins, ischemia, and the generation of cytotoxic reactive oxygen species after reperfusion [16].

Ferritin is a marker of iron stores and inflammation [19]. Serum ferritin is a widely recognized indicator of inflammation; however, its role in the inflammatory cycle and whether it merely reflects or induces inflammation remain unknown. The premise is that serum ferritin is produced by injured cells and hence serves as a marker of cellular damage [23].

Hemoglobin reflects oxygen-carrying capacity and erythropoiesis. Hemoglobin plays a crucial role in both the immune response to infections and the physiological processes of leukocytes [20].

Despite the increasing amount of information about the predictive significance of LDH, ferritin, and hemoglobin in adult sepsis, there is still a limited understanding of their effectiveness in the diagnosis of newborn sepsis.

The aim of this study is to assess the predictive usefulness of these biomarkers in predicting the onset of newborn sepsis and their potential utility in influencing medical management decisions.

## 2. Materials and Methods

### 2.1. Study Design and Settings

A case–control study was conducted at a single medical center to evaluate the potential of LDH, ferritin, and hemoglobin as biomarkers for the identification of sepsis. The present investigation was carried out on neonates who were hospitalized in the clinical unit of the Clinical Hospital “Louis Turcanu”, Timisoara, Romania, during the period spanning from 1 October 2018 to 1 July 2023. This research study received approval from the Ethics Committee of the University of Medicine and Pharmacy “Victor Babes” Timisoara, with the reference number 22726/17/11/2021.

### 2.2. Study Design and Settings

A nearest-neighbor matching method was used to select a control group of newborns. Participants were divided into two groups: sepsis-positive (cases) and sepsis-negative (control). Inclusion criteria for the group of cases were as follows: (1) newborns admitted to the Children’s Clinical Hospital “Louis Turcanu” Timisoara during the study period; (2) informed consent of the legal representative of the newborn; (3) positive diagnosis of sepsis; (4) complete laboratory analysis data. The control group included newborns who met the following criteria: (1) newborns admitted to the Children’s Hospital “Louis Turcanu” Timisoara during the study period; (2) informed consent of the newborn’s legal representative at admission; (3) complete data from laboratory tests.

Exclusion criteria were as follows: (1) non-obvious consent to use data from the legal representative; (2) missing data; (3) presence of subchorionic hemorrhage during pregnancy.

### 2.3. Variables, Data Sources, and Measurement

Three researchers collected information from the neonates’ digital medical records using a predetermined approach. The clinical data and laboratory tests collected were as follows: gestational age (GA) at delivery, infant weight in grams, neonate sex, type of birth (c-section or natural birth), pregnancy complications (fetal growth restriction, pregnancy-induced hypertension, gestational diabetes, premature rupture of membranes, preterm birth), APGAR score at 1 min, and maternal characteristics (parity, urinary tract infections during pregnancy or peripartum, positive cervical culture).

At admission, venous blood samples were taken from every baby for laboratory testing. The parameters for complete blood counts (CBCs) were measured using a SYSMEX XN 1000 (Sysmex Corporation, Kobe, Japan) and Sysmex XN-550 (Sysmex Corporation, Kobe, Japan). Cobas Integra 400 plus (Roche Diagnostics Ltd., CH-6343, Rotkreuz, Switzerland) was utilized to perform plasma LDH testing. Serum ferritin was also assayed using Cobas Integra 400 plus (Roche Diagnostics Ltd., CH-6343, Rotkreuz, Switzerland).

The outcome of interest was a diagnosis of sepsis.

A diagnosis of sepsis was established by positive blood culture evidence. Blood culture required the collection of a blood sample volume of 1 mL in preterm infants weighing less than 1 kg and 1–2 mL in infants weighing between 1.1 and 4 kg. The blood sample was inseminated in a BacTALERT PF (Organon-Teknika Corp., Durham, NC, USA) blood culture flask and placed on the BacTALERT system (Organon-Teknika Corp., Durham, NC, USA) within less than 2 h of collection.

### 2.4. Statistical Analysis

In order to conduct statistical calculations, the SPSS 20.0 software (SPSS Inc., Chicago, IL, USA) was utilized. A comparison was made between continuous variables, which are represented by medians and interquartile ranges (IQR), using the Mann–Whitney test. Using Fisher’s exact test, categorical variables expressed as counts and percentages were compared. In order to evaluate the predictive performance of the biomarkers in the diagnosis of sepsis, the area under the curve alongside the corresponding receiver operating characteristic curve were calculated. By employing Youden’s index, the optimal cut-off values for biomarkers were ascertained. The estimation of the association was conducted using binominal logistic regression.

## 3. Results

### 3.1. Baseline Characteristiscs

This study included a total of 86 neonates, 51 (59.3%) of whom had a confirmed diagnosis of sepsis and 35 (40.7%) who did not. Out of the neonates who were diagnosed with sepsis, 25 (49.0%) had late-onset sepsis (LOS) and 26 (51.0%) had early-onset sepsis (EOS).

This study found no significant differences in gestational age, infant weight at birth, infant sex, cesarean birth, preterm birth, fetal growth restriction, APGAR score at 1 min, or fetal anemia between neonates with and without sepsis. 

Neonates with sepsis had a higher incidence of cesarean delivery (39.5%) compared to those without sepsis (20.0%), and this difference was statistically significant (*p* = 0.003) (Table 1). Also, preterm birth and fetal growth restriction occurred in the majority of sepsis neonates, but this was not statistically significant.

This study found that newborns without sepsis had higher levels of neonatal hemoglobin (13.20 g/dL) compared to neonates with sepsis (11.90 g/dL), with a borderline *p*-value (0.05).

Furthermore, neonates with sepsis had significantly higher levels of neonatal serum ferritin (median 467 ng/mL) in comparison to those without sepsis (median 167 ng/mL) (*p* < 0.001). Also, neonates with sepsis had considerably higher levels of lactate dehydrogenase compared to those without sepsis (median 847 U/L vs. 498 U/L) (*p* < 0.001) (Table 1).

### 3.2. Baseline Characteristiscs

**Figure 1:**
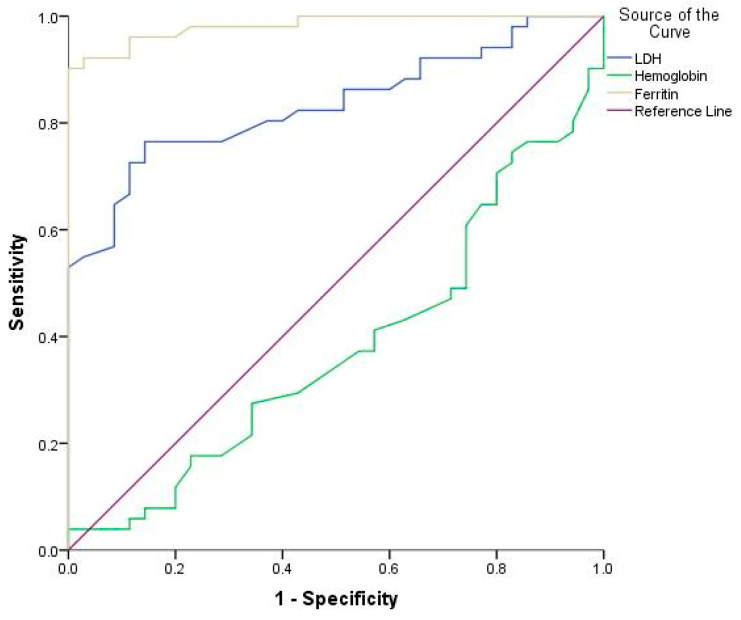
Receiver operating characteristic (ROC) curves of LDH, ferritin, and hemoglobin in diagnosis neonatal sepsis.

The diagnostic efficacy of hemoglobin, ferritin, and lactate dehydrogenase (LDH) in predicting neonatal sepsis was evaluated using receiver operating characteristic (ROC) analysis (Figure 1).

For each biomarker, the area under the curve (AUC), 95% confidence interval (CI), appropriate cut-off values, Youden’s index, and the corresponding statistical significance were assessed (Table 2). The AUC-ROC value for hemoglobin was determined to be 0.380, suggesting a poor capacity for distinguishing neonatal sepsis. It was determined that the ideal threshold value was 16.85 g/dL, and the corresponding Youden’s index was 0.039. While not statistically significant (*p* = 0.059), the specificity of hemoglobin was found to be 97.1%, while its sensitivity was comparatively low, at 39.5% (Table 2).

In contrast, ferritin demonstrated an excellent discriminatory capability, with an area under the curve of 0.982. Ferritin had an ideal cut-off of 248.5 ng/mL, which resulted in a Youden’s index of 0.893. This biomarker exhibited substantial specificity (97.1%) and sensitivity (92.2%) in its ability to diagnose neonatal sepsis, and these results were statistically significant (*p* < 0.001). Similarly, LDH exhibited excellent discriminatory power, as evidenced by its AUC of 0.834. It was determined that the optimal LDH cut-off value is 589 U/L, with a Youden’s index of 0.622. The predictive accuracy of LDH for neonatal sepsis was 85.7% in terms of specificity and 76.5% in terms of sensitivity, both of which were statistically significant (*p* < 0.001) (Table 2).

A significant positive correlation was observed between ferritin levels and the probability of neonatal sepsis, as determined by univariate binomial logistic regression (OR = 1.045, *p* = 0.001). A unit increase in ferritin concentration was associated with a 4.5% increase in the probability of neonatal sepsis (Table 3).

Likewise, a statistically significant positive correlation was observed between LDH levels and the probability of neonatal sepsis (OR = 1.007, *p* < 0.001). There was a 0.7% increase in the likelihood of neonatal sepsis for every unit increase in LDH concentration (Table 3).

Multivariate binomial logistic regression analysis was employed to investigate the association between biomarkers and the probability of neonatal sepsis, while accounting for any confounding factors.

The likelihood of neonatal sepsis continued to be significantly correlated with ferritin levels (B = 0.066, *p* = 0.01). The adjusted odds ratio (aOR) for ferritin was 1.069, indicating that the risk of neonatal sepsis increased by 6.9% for each unit increase in ferritin concentration. Similarly, after adjusting, LDH concentrations remained significantly positively correlated with the probability of neonatal sepsis (B = 0.008, *p* < 0.001). The aOR for LDH was 1.008, indicating that the odds of neonatal sepsis increased by 0.8% for every unit increase in LDH concentration (Table 4). 

On the contrary, when confounding variables were accounted for, no statistically significant correlation was observed between hemoglobin levels and the probability of neonatal sepsis (B = −0.136, *p* = 0.25) (Table 4).

Furthermore, using predefined cut-off values, multivariate logistic regression showed that neonates with ferritin levels greater than 248.5 ng/mL had a substantially increased likelihood of neonatal sepsis (aOR= 1238.5, *p* < 0.001). In the same vein, neonates whose LDH levels exceeded 589 U/L had a substantially increased probability of developing neonatal sepsis, with an aOR of 16.09 (*p* < 0.001) (Table 5). 

On the other hand, neonates categorized as anemic, defined as hemoglobin levels below 13.5 g/dL, did not demonstrate an increase in the probability of neonatal sepsis that was statistically significant (aOR= 1.91, *p* = 0.22) (Table 5).

## 4. Discussion

The data presented in this study pertain to 86 neonates who were admitted to the Children’s Clinical Hospital “Louis Turcanu” in Timisoara, Romania. Neonatal sepsis was identified in 55 of these infants. There were no statistically significant variations observed between neonates who had sepsis and those who did not with regard to gestational age, infant weight, cesarean delivery, preterm birth, fetal growth restriction, APGAR score, or fetal anemia. Conversely, a systematic review revealed that the risk of neonatal sepsis was 3.36 times greater in preterm infants compared to term infants [24]. However, there was a 3.25-fold increased risk of early-onset neonatal sepsis for term neonates delivered via C-section compared to vaginal delivery, according to Tri Utomo et al. [25]. Furthermore, with reference to the correlation between neonatal sepsis and baby weight, existing data indicates that newborns with a very low birth weight (less than 1500 g) are very susceptible to both early- and late-onset sepsis [26].

The present study aimed to examine the significance of biomarkers in the diagnosis of newborn sepsis. The primary findings of our study indicate noteworthy correlations between heightened levels of ferritin and LDH and the likelihood of infant sepsis, even after accounting for potential confounding factors. In particular, infants with ferritin levels above 248.5 ng/mL demonstrated a significant elevation in the probability of developing sepsis. Likewise, higher levels of LDH (>589 U/L) were strongly linked to a higher probability of sepsis, with an odds ratio of 16.09. In contrast, there was no statistically significant correlation between anemia (hemoglobin < 13.5 g/dL) and newborn sepsis.

Van Anh et al. demonstrated that children with early-onset newborn sepsis exhibited notably elevated LDH levels compared to those without early-onset neonatal sepsis, which aligns with our findings [27]. Additionally, it has been shown that the level of LDH serves as a predictive indicator for severe sickness in newborns [15].

Regarding ferritin, research has revealed that higher serum levels are connected with a higher risk of death in critical patients with sepsis in the adult population [19]. However, in the adult population, ferritin levels exceeding 300 ng/mL had a sensitivity of 60% and a specificity of 70% in detecting sepsis [28]. Furthermore, the correlation between ferritin levels and control variables, including birth weight and its z-score, as well as those linked to infection and inflammation, was validated in the neonatal population [29]. Additionally, serum ferritin seems to be a more accurate indicator of neonatal sepsis mortality risk than CRP [30]. A study using a threshold of 300 ng/mL or more as a marker of sepsis showed a sensitivity of 60% and a specificity of 70% [28], lower values than those detected in our study, where for a threshold of 248.5 ng/mL, the sensitivity was 92.2% and the specificity 97.1%.

In relation to the utilization of ferritin as a prognostic indicator for unfavorable outcomes in pediatric patients who were septic, Shaikh et al. conducted research which revealed that the median serum ferritin level was significantly elevated in non-survivors at 48 h of admission, as opposed to survivors. Furthermore, the study identified a significant correlation between serum ferritin and the pediatric Sequential Organ Failure Assessment (pSOFA) score and Pediatric Risk of Mortality III (PRISM III) [31]. Also, in a study of 101 newborns, Mittal et al. showed that serum ferritin with a value above 200 ng/mL had a sensitivity of 74.4% and a specificity of 54.5% in predicting mortality among neonates with sepsis, thus being considered a better predictor than C-reactive protein [30]. Also, a study conducted by Sucianto et al. shown that ferritin levels had a sensitivity of 87.5% and a specificity of 75% in predicting death in septic children. The area under the curve (AUC) was found to be 0.760, with the optimal cut-off value for ferritin levels being 975 ng/mL [32].

Nevertheless, among women experiencing early rupture of membranes before 32 weeks of gestation, there was a notable correlation between plasma ferritin levels and newborn sepsis [33]. Furthermore, our study did not show an association of anemia with neonatal sepsis. Conversely, anemia in neonates was discovered to be correlated with elevated CRP levels, sepsis in the neonate, and inadequate iron consumption by the mother according to Mansoor Aslamzai et al. [34]. Additionally, anemia is a prevalent characteristic observed in the adult population during sepsis, resulting from iatrogenic blood loss, reduction in serum iron levels and erythropoietin synthesis, and a decrease in the lifespan of erythrocytes [35]. Regarding the neonatal population, a study by Adane et al. showed that the prevalence of anemia in neonates with sepsis is 49% [36].

Regarding the diagnostic significance of hemoglobin as a marker for newborn sepsis, Minichil Worku et al. demonstrated that it has a sensitivity of 43.2%, which is similar to the sensitivity of our study (39.5%). The specificity seen in the study conducted by Minichil Worku et al. was 80%, which is lower than the sensitivity shown in our investigation (97.1%) [37]. Moreover, according to the same study, a hemoglobin level below 16.86 g/dL has a 68% sensitivity and a specificity of 53.6% in the diagnosis of sepsis [37].

Regarding anemia as a predictor of poor outcomes, in their study, Na Cai et al. showed that a reduction in infant hemoglobin levels was a significant indicator of complications in septic neonates with late-onset sepsis (LOS). The receiver operating characteristic (ROC) study of LOS demonstrated an area under the curve (AUC) of 0.807. This indicates that a reduction in Hb levels may serve as a predictive factor for complications for neonates with LOS [38].

Despite the significant results of our research, it is essential to acknowledge several limitations. This research study used an observational design, which consequently limits our ability to establish causal relationships between neonatal sepsis and biomarker blood levels. In addition, the research that we conducted may be affected by selection bias as a result of relying exclusively on neonates who were admitted to hospital and possessed available biomarker data. The consequence of this could have been the exclusion of those who were not tested. In addition, it is essential to recognize that the retrospective nature of our research, which relied on inadequate or inaccurate medical records to collect data, could potentially introduce information bias.

The interpretation of the results of this study should be cautious, given the inherent limitations of this study. Although our results indicate that elevated ferritin and LDH levels may be useful biomarkers in predicting neonatal sepsis, further prospective research is needed to confirm these correlations and establish a causal relationship.

## 5. Conclusions

The research in this paper highlights the potential predictive value of ferritin and LDH concentrations as biomarkers associated with neonatal sepsis. The results show that elevated ferritin and LDH levels were significantly associated with an increased risk of sepsis in neonates. Based on these findings, ferritin and LDH may function as useful biomarkers to detect neonates who are particularly susceptible to sepsis. This would allow clinicians to implement prompt interventions that can improve patient outcomes. However, in our study, hemoglobin was not demonstrated to be a potential marker of sepsis diagnosis due to having a discriminatory power below the limit.

## Figures and Tables

**Table 1:** Baseline and clinical characteristics of the 86 newborns included in this study.

Variable	Total N = 86	Sepsis N= 51	No Sepsis N = 35	*p*-Value
GA at delivery	35 [5]	34 [6]	35 [6]	0.22
Infant weight (grams)	2325 [1082.5]	2380 [1180]	2100.0 [890]	0.69
Male sex	58 (67.4%)	39 (76.5%)	19 (54.3%)	0.18
Cesarean birth	34 (39.5%)	27 (52.9%)	7 (20.0%)	0.003
Preterm birth	70 (81.4%)	45 (88.2%)	25 (71.4%)	0.08
Fetal growth restriction	32 (37.2%)	15 (29.4%)	17 (48.6%%)	0.11
APGAR score at 1 min	7 [2]	7 [3]	7 [1]	0.63
Neonatal anemia (Hb < 13.5 g/dL)	54 (62.8%)	35 (68.6%)	19 (54.3%)	0.25
Neonatal hemoglobin	12.6 [3.6]	11.90 [3.40]	13.20 [3.10]	0.05
Neonatal serum ferritin	278.5 [305.25]	467 [200]	167 [74]	<0.001
LDH	589 [401]	847 [574]	498 [143]	<0.001
**Maternal characteristics**				
Parity	2 [2]	2 [3]	2 [2]	0.87
Pregnancy-induced hypertension	3 (3.5%)	3 (6.0%)	-	NA
Gestational diabetes	1 (1.2%)	-	1 (2.9%)	NA
Premature rupture of membranes	20 (23.3%)	17 (33.3%)	3 (8.6%)	0.009
Urinary tract infections	30 (34.9%)	30 (58.8%)	-	NA
Positive cervical culture	8 (9.3%)	8 (15.7%)	-	NA

Continuous variables are expressed as medians [interquartile intervals]. Categorical variables are expressed as absolute counts (percentages). Hb = hemoglobin; LDH = lactate dehydrogenase.

**Table 2:** Receiver operating characteristics (ROC) curves, diagnostic accuracy of biomarkers, and optimal cut-off.

Variable	AUC	95%CI		Cut-Off	Youden	*p*-Value	Sensitivity	Specificity
		Lower	Upper					
Hemoglobin	0.380	0.260	0.499	16.85	0.039	0.059	39.5%	97.1%
Ferritin	0.982	0.961	1.00	248.5	0.893	<0.001	92.2%	97.1%
LDH	0.834	0.750	0.918	589	0.622	<0.001	76.5%	85.7%

AUC = area under the receiver operating characteristic (ROC) curve; CI = confidence interval; LDH = lactate dehydrogenase.

**Table 3:** The likelihood of neonatal sepsis according to univariate binomial logistic regression.

Biomarker	B	SE	*p*-Value	OR	95%CI	
					Lower	Upper
Ferritin	0.044	0.013	0.001	1.045	1.018	1.073
LDH	0.007	0.002	<0.001	1.007	1.003	1.011
Hemoglobin	−0.183	0.103	0.07	0.833	0.680	1.019

B = log odds ratios (the logistic regression coefficients); SE = standard error; OR = odds ratio; CI = confidence interval; LDH = lactate dehydrogenase.

**Table 4:** The likelihood of neonatal sepsis according to multivariate binomial logistic regression.

Biomarker	B	SE	*p*-Value	aOR	95%CI	
					Lower	Upper
Ferritin	0.066	0.028	0.01	1.069	1.011	1.130
LDH	0.008	0.002	<0.001	1.008	1.003	1.012
Hemoglobin	−0.136	0.120	0.25	0.873	0.690	1.103

Confounding variables include the following: gestational age, pregnancy complications (fetal growth restriction, pregnancy-induced hypertension, gestational diabetes, premature rupture of membranes, preterm birth), and maternal characteristics (parity, urinary tract infections during pregnancy or peripartum, positive cervical culture). B = log odds ratios (logistic regression coefficients); SE = standard error; aOR = adjusted odds ratio; CI = confidence interval; LDH = lactate dehydrogenase.

**Table 5:** The likelihood of neonatal sepsis according to multivariate binomial logistic regression, using predefined cut-off values.

Biomarker	B	SE	*p*-Value	OR	95%CI	
					Lower	Upper
Ferritin > 248.5	7.122	1.809	<0.001	1238.5	35.723	42,940.5
LDH > 589	2.778	0.744	<0.001	16.09	3.746	69.130
Anemia (HB < 13.5)	0.649	0.535	0.22	1.91	0.671	5.457

Confounding variables include the following: gestational age, pregnancy complications (fetal growth restriction, pregnancy-induced hypertension, gestational diabetes, premature rupture of membranes, preterm birth), and maternal characteristics (parity, urinary tract infections during pregnancy or peripartum, positive cervical culture). B = log odds ratios (logistic regression coefficients); SE = standard error; OR = odds ratio; CI = confidence interval; LDH = lactate dehydrogenase.

## Data Availability

The data sets used and/or analyzed during the present study are available from the first author on reasonable request.
